# Dietary Diversity Is Associated With Memory Status in Chinese Adults: A Prospective Study

**DOI:** 10.3389/fnagi.2020.580760

**Published:** 2020-09-02

**Authors:** Jian Zhang, Ai Zhao, Wei Wu, Chenlu Yang, Zhongxia Ren, Meichen Wang, Peiyu Wang, Yumei Zhang

**Affiliations:** ^1^Department of Nutrition and Food Hygiene, School of Public Health, Peking University, Beijing, China; ^2^Vanke School of Public Health, Tsinghua University, Beijing, China; ^3^Department of Social Medicine and Health Education, School of Public Health, Peking University, Beijing, China

**Keywords:** memory status, memory decline, dietary diversity, adults, prospective study

## Abstract

**Background and Aim:**

Subjective memory complaints are common in elderly people. Nutrition plays an important role in keeping brain health, however, the evidence on dietary diversity and subjective memory status is limited. This study aimed to investigate the effect of dietary diversity score (DDS) on memory status in Chinese adults in a prospective cohort study.

**Methods:**

Data of the China Health and Nutrition Survey was used in this study. A total of 4356 participants aged 50 years or older were enrolled in the analysis. DDS was calculated based on the dietary recall data collected in the wave of 2011. Information on self-report memory status (OK, good, or bad) and memory change in the past 12 months (stayed the same, improved, or deteriorated) were obtained from the wave of 2015. A memory score was calculated based on a subset of items of the Telephone Interview for Cognitive Status-modified. Multinomial logistic regression models were used to estimate the associations of DDS with memory status and memory change, and linear regression models were carried out to estimate the association between DDS and memory score.

**Results:**

In the study population, the percentages of participants who thought their memory was OK, bad, and good were 43.3, 24.3, and 32.4%, respectively. There were 1.4% of participants reported memory improvement in the past 12 months and 47.2% reported memory decline. Average memory score among participants was 12.8 ± 6.1. Compared with participants who thought their memory was OK, a higher DDS was associated with self-reported good memory (Odds Ratio [OR] 1.15, 95%CI 1.07–1.24) and inversely associated with bad memory (OR 0.82, 95%CI 0.75–0.89). In subgroup analysis, however, in participants aged 65 years and above, the association between DDS and self-reported good memory was insignificant (OR 1.09, 95%CI 0.94–1.25). Compared with participants whose memory stayed the same, higher DDS was inversely associated with memory decline (OR 0.85, 95%CI 0.80–0.91). Besides, higher DDS was associated with higher memory score (β 0.74, 95%CI 0.56–0.91).

**Conclusion:**

This study revealed that higher DDS was associated with better memory status and was inversely associated with self-reported memory decline in Chinese adults.

## Introduction

Subjective memory complaints are common in elderly people (Caramelli and Beato, 2008), which affects the quality of life of elderly people negatively. Memory complaint is not only an age-related phenomenon but also an early signal of Alzheimer’s disease and dementia (Jonker et al., 2000; Peters et al., 2019). In community-dwelling elderly individuals, there is a 25–50% prevalence rate of memory complaints (Jonker et al., 2000). In 2015, people over 60 years constituted 12% of the world’s population, and the population is aging at a faster pace than the past (World Health Organization [WHO], 2018). Memory loss has become an important public health issue and a social concern (Centers for Disease Control and Prevention, 2019; World Health Organization [WHO], 2019), thus actions to prevent early memory decline will be beneficial to both the quality of individual life and the burdens of society.

Lifestyle intervention as a cost-effective way to prevent some age-related health diseases has been recognized by increasingly more people (Katz et al., 2018). Healthy diets can delay dementia progression and reduce the risk of Alzheimer’s disease in the elderly (George and Reddy, 2019). A cohort study in Australia showed high consumption of fruit, vegetables, and protein-rich food was associated with lower odds of self-reported memory loss (Xu et al., 2020). Another study in Chinese showed higher fish consumption was associated with a slower decline in memory in adults aged 65 years and above (Qin et al., 2014). Besides, a cohort study revealed that higher adherence to the Mediterranean diet was inversely associated with poor subjective cognitive function (Bhushan et al., 2018). Several studies showed that high dietary diversity decreased the risk of cognitive decline (Clausen et al., 2005; Otsuka et al., 2017). However, evidence on dietary diversity and memory status from large-scale, prospective studies were limited.

Dietary diversity has long been recognized as a key element of diet. As a tool to assess both nutrient adequacy of individual and food security of household (Kennedy et al., 2011; Salehi-Abargouei et al., 2016), dietary diversity score (DDS) has been widely used in different populations (Salehi-Abargouei et al., 2016). Several age-related diseases, including diabetes (Conklin et al., 2016) and hypertension (Kapoor et al., 2018), are reported to be inversely associated with DDS. As the elderly people usually experience accelerated mobility decreasing, degeneration of the digestive system, and decline in appetite (Favaro-Moreira et al., 2016; Zhao et al., 2019), they may face higher risks of nutrient deficiency (Ahmed and Haboubi, 2010), which may further generate negative effects on memory. In this study, we investigated the effect of dietary diversity on memory status in Chinese adults aged 50 years and above.

## Materials and Methods

### Study Population

**FIGURE 1:**
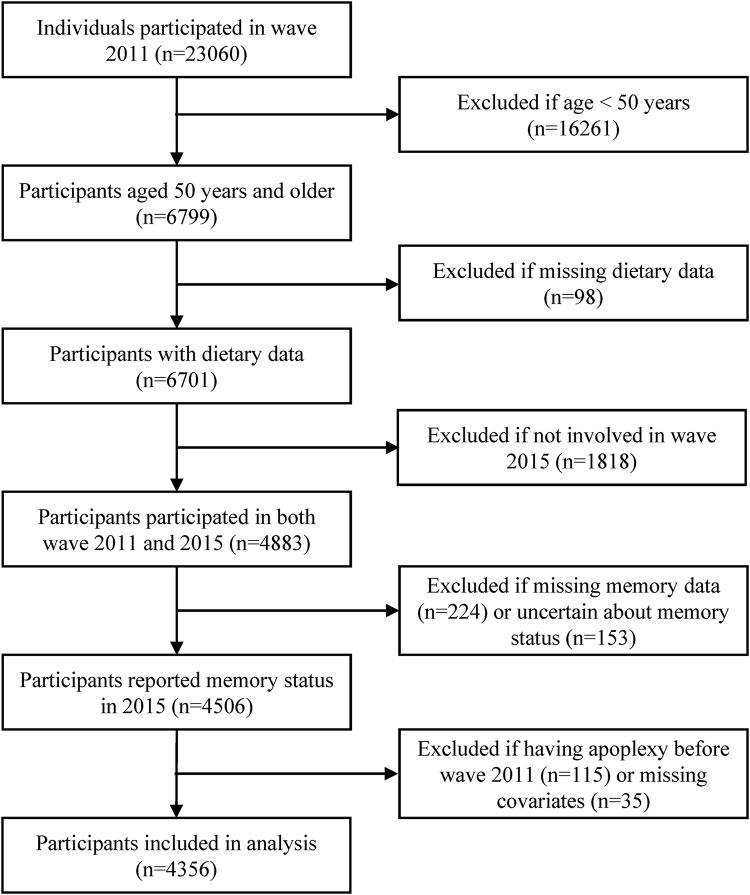
Flow chart of sample selection.

Data was obtained from the China Health and Nutrition Survey (CHNS). The CHNS is a national-wide, dynamic cohort study initialed in 1989, aiming to understand the health and nutrition status of Chinese and how they are affected by social and economic transformation. Details about the CHNS has already been published (Zhang et al., 2014). Data collected in the wave of 2011 and 2015 were used in this study to estimate the dietary diversity and to obtain information on memory status respectively. The inclusion criteria of our study included aged 50 years and above in the wave of 2011, participated in the dietary survey in the wave of 2011, participated in the follow-up survey in wave 2015. The exclusion criteria included diagnosed with apoplexy, uncertain about one’s memory status, and having missing values on covariates. In the end, 4356 participants from 12 areas of China (Beijing, Liaoning, Heilongjiang, Shanxi, Jiangsu, Shandong, Henan, Hubei, Hunan, Guangxi, Guizhou, and Chongqing) were included in our analyses (Figure 1).

### Dietary Survey and Dietary Diversity Score

Dietary intake was assessed by individual dietary recall for 3 consecutive days in combination with family food weight inventory. Participants were asked to report all the foods and beverages they consumed during a 24-h period. More details about the dietary survey process have been described elsewhere (Zhai et al., 1996). The present study used dietary data collected in the wave of 2011. All food items were divided into eight food categories (cereals and tubers, vegetables, fruits, meat, soybeans and nuts, eggs, aquatic products, and milk and dairy products). If one participant consumed any food from a certain food category in the past 24 h, then he would get one point for that food category, with a total score of eight. Average daily scores were calculated for each participant. Besides, participants’ daily energy and nutrient intakes were estimated based on the China food composition databases (Yang, 2005; Yang et al., 2009).

### Ascertain of Memory Status

In the wave of 2015, participants’ memory status and memory change in the past 12 months were surveyed. For memory status, participants were asked “How is your memory?” (very good, good, OK, bad, very bad, or unknown). We classified participants’ memory status into good (very good/good), OK, and bad (very bad/bad) for further analysis. For memory change over last year, participants were asked “In the past 12 months, how has your memory changed?” (improved, stayed the same, deteriorated, or unknown). Individuals who were unknown about their memory status or memory change were excluded from the analysis.

Besides, a subset from the Telephone Interview for Cognitive Status-modified (Brandt et al., 1988; van den Berg et al., 2012) was used to determine participants’ memory function. The test items included immediate and delayed free-recall test (ten words) and the Serial 7s test. These tests assess participants’ verbal memory and working memory (van den Berg et al., 2012). Details about the tests were published elsewhere (Herzog and Wallace, 1997; Qin et al., 2014). Participants whose answer was “unknown” did not get a score for the test item. Total memory scores rank from 0 to 25. 98.6% of participants took this test.

### Covariates

Covariates were obtained from the wave of 2011. Covariates of sociodemographic characteristics and lifestyle behaviors used in this study included age (continuous), gender (men/women), living region (southern/northern China), education level (primary school or lower, lower middle school, or middle school or above), alcohol consumption (never drink, <3, or ≥3 times a week), smoking status (never smoke, used to smoke, currently smoke), and annual per capita household income. 21.9% of participants had missing data on income information, and missing values of annual per capita household income were replaced by the medians of each survey site. Income was classified into as low, middle, or high, corresponding to annual per capita household income 10,000 and below, 10,000–20,000, and over 20,000 RMB, respectively. Besides, medical history used in this analysis included previously diagnosed apoplexy (yes/no), diabetes (yes/no), myocardial infarction (yes/no), and hypertension (yes/no). Since blood pressures were measure in the wave of 2011 (99.5% of participants took this test), participants who had been diagnosed with hypertension or whose systolic blood pressure ≥ 140 mmHg and/or diastolic blood pressure ≥90 mmHg were all regarded as hypertensive patients (Hua et al., 2019).

### Statistics

Normally distributed continuous variables were presented with Means and SDs; otherwise, medians and quartiles were used. Categorical variables were presented with percentages. Differences across groups were compared with one-way ANOVA or Chi-square tests for normally distributed continuous and categorical variables, respectively. Tests for linear trend of nutrient intakes across DDS categories were performed by assigning the midpoint values of DDS categories and treating the variable as continuous in a separate regression model, prior to that, values of nutrient intakes were transformed to log to reach normality. Multinomial logistic regression models were conducted to investigate the association of DDS scores with self-reported memory status (OK, good, or bad; participants whose memory was OK as the comparison group) and memory change in the past 12 months (stayed the same, improved, or deteriorated; participants whose memory stayed the same as the comparison group). Linear regression models were carried out to explore the association between DDS and memory scores. Multivariate models were conducted, and factors with *P* < 0.05 in the univariate analyses were included. In the first model, covariate including age, living region, education level, and income, were adjusted. In the second model, smoking status, alcohol consumption, and history of diabetes were additionally adjusted. To confirm the robustness of our findings, sensitivity analyses were conducted by (1) additional adjustment of gender, history of infarction, and history of hypertension; (2) excluding participants whose income information were missing at baseline. We also did subgroup analyses according to gender (men or women) and age (<65 or ≥65 years). Statistics were conducted in R 4.0.2. The multinomial logistic regressions were conducted with R package nnet (Venables and Ripley, 2002). All *P*-values were two-sided, and statistical significance was defined as *P* < 0.05.

## Results

### Memory Status

A total of 4356 participants were included in our analysis, with an average age of (61.9 ± 7.9) years. The percentages of participants who thought their memory was OK, bad, and good were 43.3, 24.3, and 32.4%, respectively. There were 1.4% of participants reported memory improvement in the past 12 months and 47.2% reported memory decline. The average memory score among the participants was 12.8 ± 6.1.

### DDS and Its Distribution Among Different General Characteristics

Average DDS in participants was 4.09 ± 1.13. Table 1 showed the characteristics of participants across DDS categories. Participants with higher DDS were more likely to be relatively younger, living in southern China, having higher education and income, drinking less, never smoking, and having histories of diabetes. Men and women had similar DDS. Proportions of hypertension or myocardial infarction were similar in participants among different DDS categories.

**TABLE 1:** General characteristics of participants according to DDS categories.

Variables	DDS categories				*P*
	(0,2]	(2,4]	(4,6]	(6,8]	
Number of participants	157	2246	1762	191	
Age (years)	65.4 ± 8.5	62.0 ± 7.9	61.4 ± 7.8	62.2 ± 8.1	< 0.001
**Gender**					
Men	45.9	46.5	48.0	40.8	0.281
Women	54.1	53.5	52.0	59.2	
**Living region**					
Southern China	48.4	65.1	61.5	71.2	< 0.001
Northern China	51.6	34.9	38.5	28.8	
**Education**					
Primary school or lower	83.4	64.9	39.6	16.8	< 0.001
Lower middle school	11.5	22.3	30.7	24.1	
Middle school or above	5.1	12.8	29.7	59.2	
**Income**					
Low	74.5	53.2	23.8	3.1	< 0.001
Middle	21.7	33.5	36.1	24.1	
High	3.8	13.3	40.1	72.8	
**Alcohol consumption**					
Never drink	71.3	70.2	65.6	69.1	0.002
<3 times a week	15.9	13.0	15.9	19.9	
≥3 times a week	12.7	16.8	18.4	11.0	
**Smoking status**					
Never smoke	65.6	65.8	68.0	82.7	< 0.001
Used to smoke	7.6	5.3	6.4	4.7	
Currently smoke	26.8	28.9	25.6	12.6	
**Hypertension**					
No	59.9	58.4	57.7	56.5	0.897
Yes	40.1	41.6	42.3	43.5	
**Diabetes**					
No	98.7	95.3	92.5	92.7	< 0.001
Yes	1.3	4.7	7.5	7.3	
**Myocardial infarction^*a*^**					
No	98.7	99.0	98.9	99.5	0.882
Yes	1.3	1.0	1.1	0.5	

**Values are Means ± SDs for continuous variables and percentages for categorical variables unless stated otherwise. Differences across groups were compared by ANOVA and Chi-square tests for continuous and categorical variables, respectively. DDS, dietary diversity score.*^*a*^One missing value.*

### Energy and Nutrient Intakes

Table 2 shows that participants in higher DDS categories had higher intakes of energy, protein, fat, dietary fiber, cholesterol, and most micronutrients (e.g., vitamin A, vitamin C, calcium). However, a negative trend was observed between DDS and intakes of carbohydrate, sodium, and manganese.

**TABLE 2:** Energy and nutrients intake according to DDS categories.

	DDS categories				*P* for trend
	(0, 2]	(2, 4]	(4, 6]	(6, 8]	
Energy (kcal)	1540.5(1249.8,2072.8)	1788.2(1386.1,2281.2)	1915.0(1566.6,2390.8)	1956.4(1637.1,2324.8)	<0.001
Protein (g)	50.5(44.8,57.6)	58.7(50.7,68.7)	68.3(59.5,80.0)	82.3(72.2,93.7)	<0.001
Fat (g)	41.6(29.6,65.1)	74.3(56.5,93.8)	82.1(67.1,98.1)	89.2(74.4,100.0)	<0.001
Carbohydrate (g)	358.3(304.2,389.0)	281.5(234.3,324.4)	253.9(214.0,289.3)	236.3(201.5,265.1)	<0.001
Dietary fiber (g)	13.6(10.0,17.3)	12.2(8.9,16.4)	12.7(9.5,16.9)	14.3(11.4,19.2)	<0.001
Cholesterol (mg)	0.1(0.0,5.6)	163.2(76.7,299.7)	329.4(203.2,486.7)	512.7(360.8,651.5)	<0.001
Vitamin A (ugRE)	207.7(83.9,402.7)	334.4(189.4,598.5)	436.9(286.7,708.3)	639.2(413.2,893.6)	<0.001
Thiamin (mg)	0.8(0.6,1.0)	0.8(0.7,1.0)	0.8(0.7,1.0)	0.9(0.8,1.2)	<0.001
Riboflavin (mg)	0.5(0.4,0.6)	0.6(0.5,0.8)	0.8(0.7,1.0)	1.1(1.0,1.3)	<0.001
Niacin (mg)	9.9(6.4,12.5)	13.0(10.0,17.3)	14.3(11.4,17.8)	16.3(13.7,19.2)	<0.001
Vitamin C (mg)	69.0(33.8,100.8)	69.7(41.2,107.7)	78.0(50.9,114.9)	106.1(77.5,146.0)	<0.001
Vitamin E (mg)	27.3(16.7,41.1)	30.7(20.5,43.0)	31.6(22.8,42.3)	33.3(24.9,42.9)	<0.001
Calcium (mg)	293.9(205.6,380.5)	343.8(262.0,463.3)	434.6(326.5,582.7)	688.9(540.1,870.8)	<0.001
Phosphorus (mg)	886.7(755.1,989.2)	876.0(766.4,1023.0)	977.7(850.6,1136.6)	1196.8(1078.4,1339.7)	<0.001
Potassium (mg)	1568.5(1280.9,1805.0)	1595.5(1297.2,1966.9)	1801.9(1514.4,2171.9)	2237.7(1971.6,2572.7)	<0.001
Sodium (mg)	5016.2(3399.3,7131.3)	4817.8(3421.4,6838.7)	4343.6(3216.0,5959.5)	4433.9(3297.4,5580.9)	<0.001
Magnesium (mg)	321.5(263.5,366.8)	282.1(236.0,337.7)	294.2(246.9,348.5)	331.4(300.0,387.3)	<0.001
Iron (mg)	18.0(14.9,20.8)	18.0(15.3,22.1)	19.5(16.4,23.7)	20.8(18.1,25.0)	<0.001
Zinc (mg)	8.0(6.6,9.8)	9.6(8.1,11.2)	10.4(9.0,12.0)	11.7(10.1,13.3)	<0.001
Selenium (μg)	38.3(20.5,54.5)	36.2(27.7,47.3)	45.7(36.5,59.2)	61.0(48.8,77.2)	<0.001
Copper (mg)	1.3(1.1,1.7)	1.5(1.2,1.8)	1.6(1.3,2.0)	1.9(1.5,2.4)	<0.001
Manganese (mg)	5.9(4.8,7.0)	5.3(4.2,6.3)	4.9(4.0,5.9)	4.7(3.8,5.8)	<0.001

**Values are medians and quartiles unless stated otherwise. Values of nutrient intake were expressed values per 2000 kcal, except for energy. DDS, dietary diversity score. Tests for linear trend across categories were performed by assigning the midpoint values of DDS categories and treating the variable as continuous in a linear regression model, prior to that, values of nutrient intakes were transformed to log to reach normality.**

### Association of DDS With Memory Status

Compared with participants who thought their memory was OK, higher DDS was associated with self-reported good memory and inversely associated with self-reported bad memory (Table 3). Comparing with participants whose memory stayed the same in the past 12 months, higher DDS was inversely associated with self-reported memory decline (Table 4). In addition, higher DDS was associated with higher memory scores (Table 5). The adjustment of covariates did not change the trends between DDS and outcomes. No association between DDS and memory improvement was observed (Table 4).

**TABLE 3:** Association between DDS and self-reported memory status.

	DDS categories				Continuous
	(0, 2]	(2, 4]	(4, 6]	(6, 8]	
**Good**					
Crude	Ref	3.06(1.77,5.29)***	4.30(2.48,7.44)***	5.09(2.75,9.44)***	1.26 (1.18, 1.34)***
Model 1	Ref	2.58(1.48,4.49)**	3.02(1.72,5.29)***	3.13(1.65,5.94)***	1.14 (1.07, 1.23)***
Model 2	Ref	2.57(1.48,4.48)**	3.02(1.73,5.30)***	3.24(1.71,6.15)***	1.15 (1.07, 1.24)***
**Bad**					
Crude	Ref	0.78(0.55,1.10)	0.51(0.36,0.73)***	0.27(0.15,0.49)***	0.76 (0.70, 0.81)***
Model 1	Ref	0.96(0.67,1.38)	0.72(0.49,1.05)	0.40(0.21,0.76)**	0.82 (0.75, 0.89)***
Model 2	Ref	0.95(0.66,1.37)	0.71(0.48,1.04)	0.39(0.20,0.74)**	0.82 (0.75, 0.89)***

*Values are odds ratios (95%CIs).* **P < 0.01; ***P < 0.001. *DDS, dietary diversity score; Ref, reference. Nominal logistic regression models were conducted to investigate the association between DDS and self-reported memory status (OK, good, or bad), taking the participants whose memory was OK as the comparison group. Multivariate models were adjusted for: Model 1: age (continuous), living region (southern/northern China), education level (primary school or lower, lower middle school, or middle school or above), and income (low, middle, or high); Model 2: additionally included alcohol consumption (never drink, <3, or ≥3 times a week), smoking status (never smoke, used to smoke, currently smoke), and history of diabetes (yes/no).*

**TABLE 4:** Association between DDS and self-reported memory change in the past 12 months.

	DDS categories				Continuous
	(0, 2]	(2, 4]	(4, 6]	(6, 8]	
**Improved**					
Crude	Ref	1.38(0.19,10.30)	1.57(0.21,11.66)	2.06(0.24,17.93)	1.08(0.87,1.35)
Model 1	Ref	1.46(0.19,11.11)	1.53(0.20,11.94)	1.75(0.18,16.55)	1.02(0.79,1.32)
Model 2	Ref	1.45(0.19,11.02)	1.43(0.18,11.25)	1.56(0.16,14.82)	1.00(0.77,1.29)
**Deteriorated**					
Crude	Ref	0.58(0.42,0.82)**	0.42(0.30,0.59)***	0.22(0.14,0.34)***	0.77(0.73,0.81)***
Model 1	Ref	0.74(0.52,1.05)	0.66(0.46,0.94)*	0.38(0.23,0.62)***	0.86(0.81,0.92)***
Model 2	Ref	0.73(0.51,1.03)	0.64(0.45,0.92)*	0.35(0.22,0.58)***	0.85(0.80,0.91)***

*Values are odds ratios (95%CIs).* *P < 0.05;**P < 0.01;***P < 0.001. DDS, dietary diversity score; Ref, reference. Nominal logistic regression models were conducted to investigate the association between DDS and self-reported memory change in the past 12 months (stayed the same, improved, or deteriorated), taking the participants whose memory stayed the same in the past 12 months as the comparison group. Multivariate models were adjusted for: Model 1: age (continuous), living region (southern/northern China), education level (primary school or lower, lower middle school, or middle school or above), and income (low, middle, or high); Model 2: additionally included alcohol consumption (never drink, <3, or ≥3 times a week), smoking status (never smoke, used to smoke, currently smoke), and history of diabetes (yes/no).

**TABLE 5:** Association between DDS and memory score.

	DDS categories				Continuous
	(0, 2]	(2, 4]	(4, 6]	(6, 8]	
Crude	Ref	0.74(−0.23,1.71)	2.67(1.69,3.64)***	5.66(4.39,6.93)***	1.26(1.10,1.41)***
Model 1	Ref	−0.46(−1.39,0.46)	0.54(−0.42,1.49)	2.85(1.59,4.12)***	0.73(0.55,0.90)***
Model 2	Ref	−0.44(−1.36,0.49)	0.58(−0.38,1.53)	2.96(1.69,4.22)***	0.74(0.56,0.91)***

*Values are β (95%CIs).* ***P < 0.001. DDS, dietary diversity score; Ref, reference. Memory scores was calculated based on a subset from the Telephone Interview for Cognitive Status-modified, including immediate and delayed free-recall test (10 words) and the Serial 7s test. 98.6% of participants took this test. Linear regression models were conducted to investigate the association between DDS and memory scores. Multivariate models were adjusted for: Model 1: age (continuous), living region (southern/northern China), education level (primary school or lower, lower middle school, or middle school or above), and income (low, middle, or high); Model 2: additionally included alcohol consumption (never drink, <3, or ≥3 times a week), smoking status (never smoke, used to smoke, currently smoke), and history of diabetes (yes/no).

### Sensitivity Analyses

In the sensitivity analyses, the associations of DDS with memory status, memory change, and memory score did not change after additional adjustment of gender, history of hypertension, and history of myocardial infarction (Supplementary Table 1) or excluding participants whose income information were missing at baseline (Supplementary Table 2).

### Subgroup Analyses

In the subgroup analyses by gender, the associations were consistently between men and women and did not change appreciably compared with the results of the combined population. In the subgroup analyses by age (<65, ≥65 years), higher DDS was associated with self-reported good memory in participants aged below 65 years but not in those aged 65 years and above (Supplementary Table 3).

## Discussion

To our knowledge, the present study is the first one providing prospective evidence about dietary diversity and memory status in the Chinese population. Our study found that higher DDS was associated with self-reported good memory and higher memory score and was inversely associated with self-reported bad memory and memory decline.

In this study, we observed higher DDS was associated with better memory status and inversely associated with subjective memory decline. One of the explanations for the association could be explained by the positive trend between DDS and intakes of antioxidants. In the present study, we found participants with higher DDS had higher intakes of antioxidants, such as vitamin C and vitamin E. It has been long recognized that oxidative damage might lead impairment to the brain in aged people (Head, 2009), and cohort studies showed that intakes of antioxidants were inversely associated with the risks of Alzheimer disease (Engelhart et al., 2002) and dementia (Devore et al., 2010). Another explanation is that fish is an important part of DDS, and fish provides rich high-quality n-3 long-chain polyunsaturated fatty acids (n-3 PUFA). A cohort study in Chinese found higher fish consumption was associated with a slower decline in memory in adults aged 65 years and above (Qin et al., 2014). N-3 PUFAs, such as docosahexaenoic acid (DHA), not only participate in neurogenesis, synaptogenesis, and myelination (Joffre et al., 2014) but also reduce inflammation and oxidative stress in the brain (Avramovic et al., 2012; Laye et al., 2018). A systemic review and meta-analysis showed that DHA supplementation had a beneficial effect on memory function in older adults (Yurko-Mauro et al., 2015). In addition, our study observed a negative trend between DDS and the intake of manganese. Excess manganese in the brain can be neurotoxic (Dobson et al., 2004). An observational study in the Chinese elderly found that whole blood manganese was correlated with plasma amyloid-β peptides and manganese might be involved in the progress of Alzheimer’s disease (Tong et al., 2014). Last but not least, participants with higher DDS had higher intakes of protein and most of the micronutrients, which could promote the overall health of participants and slow down memory loss.

One interesting finding in this study is, in the subgroup analysis, we observed higher DDS was positively associated with self-reported good memory in participants aged below 65 years, however, in those aged 65 years and above, the association was insignificant. We assumed that in elderly people, degenerative diseases were the main cause of memory loss. Elderly people were vulnerable to age-related, degenerative diseases, which might influence memory directly [e.g., diabetes (Gregg et al., 2000), stroke (Kuzma et al., 2018)] or indirectly [e.g., hospitalization (Wilson et al., 2012)]. However, it worth noting that, the current study also showed a relatively lower DDS in elders aged 65 years and above (4.00 ± 1.18) compared with that of participants younger than 65 years (4.13 ± 1.10, *P*_*t*__–test_ < 0.001). Nutrition interventions are needed to help elders to achieve a more diverse diet which may be beneficial to cope with the age-related memory decline.

DDS is widely used as an index of nutrient adequacy. Our study found higher DDS was associated with higher intakes of most macronutrients and micronutrients, which was consistent with previous studies (Tavakoli et al., 2016; Cano-Ibanez et al., 2019). Individuals who enjoyed higher dietary diversity had a lower intake of sodium. High dietary sodium is considered as a risk factor of hypertension (Karppanen and Mervaala, 2006), and dietary sodium related hypertension is an important public health concern in China (Liu, 2009). The prevalence of hypertension in Chinese aged 60 years and above in 2012 was 58.9% (National Health and Family Planning Commission of the People’s Republic of China, 2016), however, it is reported that 77.64% of Chinese adults aged 60 years and above consumed more salt than the recommendations of Chinese dietary guidelines in 2015 (Wang et al., 2016; Jiang et al., 2019). Improving dietary diversity might be regarded as one of the components of the strategy of hypertension prevention. Besides, we found among participants, higher DDS was associated with other healthy lifestyles, including drinking less and smoking less, which may provide comprehensive effects on memory health. Findings also have implications for nutrition education, suggesting the need of increasing overall health awareness.

The strength of this study included population-based sample and prospective design, which lend strength to interferences. There are several limitations. First, although medical histories (hypertension, diabetes, and myocardial infarction) were considered in analysis, other comorbidities and life events might also have negative impacts on participants’ memory. Second, as the dietary data were self-reported, participants might overestimate the intake of some foods and underestimate some other foods because of social desirability (Hebert, 2016). Besides, the gender difference was observed in these biases (Hebert et al., 1997). In the present study, we did subgroup analysis by gender, and the trends of DDS and memory in men and women was consistent with the whole population. Third, some participants chose “unknown” when reporting their memory status. As the survey did not further inquiry about the reason for their choices, we excluded these individuals from analysis. Fourth, assessment of subjective memory status was based on self-report not standard scales. Further studies with a validated scale to measure the memory status are welcome.

## Conclusion

Our study revealed that higher DDS was associated with better memory status and was inversely associated with self-reported memory decline in Chinese adults. Based on the findings of the present study, we proposed the recommendation of increasing diversity of diet in elderly people to promote memory health and delay memory decline progression.

## Data Availability Statement

The raw data supporting the conclusions of this article will be made available by the authors, without undue reservation.

## Ethics Statement

The studies involving human participants were reviewed and approved by the Institutional Review Boards at the University of North Carolina at Chapel Hill and the National Institute of Nutrition and Food Safety, China Centre for Disease Control and Prevention. The patients/participants provided their written informed consent to participate in this study.

## Author Contributions

JZ, AZ, and YZ: conceptualization. JZ, WW, and CY: methodology. JZ and AZ: writing original draft. ZR, MW, PW, and YZ: review and editing. All the authors have read and agreed to the published version of the manuscript.

## Conflict of Interest

The authors declare that the research was conducted in the absence of any commercial or financial relationships that could be construed as a potential conflict of interest.
