# Supplementary material for: Dietary Diversity Is Associated With Memory Status in Chinese Adults: A Prospective Study
Source: Front Aging Neurosci. 2020 Sep 2;12:580760. doi: 10.3389/fnagi.2020.580760 (PMC7494158; doi:10.3389/fnagi.2020.580760)
Supplement: Supplementary file 1 [file Table_1.docx]

**Supplementary Table 1**. Sensitivity analyses of association between DDS and memory status.*^a^*

|  | **DDS categories** | | | | **Continuous** |
| --- | --- | --- | --- | --- | --- |
|  | **(0,2]** | **(2,4]** | **(4,6]** | **(6,8]** |  |
| **Self-reported memory status (OR, 95%CI)** | | |  |  |  |
| Good | Ref | 2.60(1.49,4.53)^**^ | 3.06(1.74,5.36)^***^ | 3.28(1.72,6.22)^***^ | 1.15(1.07,1.24)^***^ |
| Bad | Ref | 0.95(0.66,1.36) | 0.70(0.48,1.03) | 0.38(0.20,0.72)^**^ | 0.81(0.75,0.88)^***^ |
| **Self-reported memory change (OR, 95%CI)** | | |  |  |  |
| Improved | Ref | 1.42(0.19,10.82) | 1.44(0.18,11.28) | 1.56(0.16,14.83) | 1.00(0.77,1.30) |
| Deteriorated | Ref | 0.73(0.51,1.03) | 0.64(0.44,0.92)^*^ | 0.35(0.22,0.57)^***^ | 0.85(0.80,0.91)^***^ |
| **Memory score (β, 95%CI)*^b^*** | | |  |  |  |
|  | Ref | -0.43(-1.35,0.50) | 0.60(-0.35,1.56) | 3.00(1.74,4.27)^***^ | 0.75(0.58,0.92)^***^ |

*^*^ P<0.05; ^**^P<0.01; ^***^P<0.001. DDS, dietary diversity score; OR, odds ratio; CI, confidence interval; Ref, reference. Nominal logistic regression models were used to estimate the association of DDS with memory status (OK, good, or bad) and memory change in the past 12 months (stayed the same, improved, or deteriorated), taking participants whose memory was OK or whose memory stayed the same as the comparison group, respectively. Linear regression models were conducted to estimate the association of DDS with memory score. Models were adjusted for age (continuous), living region (southern/ northern China), education level (primary school or lower, lower middle school, or middle school or above), income (low, middle, or high), alcohol consumption (never drink, <3, or ≥3 times a week), smoking status (never smoke, used to smoke, currently smoke), history of diabetes (yes/ no), gender (men/ women), history of hypertension (yes/ no), and history of myocardial infarction (yes/ no). ^a^ One case was excluded from analyses because of missing information on history of myocardial infarction. ^b^ Memory scores was calculated based on a subset from the Telephone Interview for Cognitive Status-modified, including immediate and delayed free-recall test (ten words) and the Serial 7s test. 98.6% of participants took the memory test.*
